# Thimerosal exposure and increased risk for diagnosed tic disorder in the United States: a case-control study

**DOI:** 10.1515/intox-2015-0011

**Published:** 2015-06

**Authors:** David A. Geier, Janet K. Kern, Brian S. Hooker, Paul G. King, Lisa K. Sykes, Kristin G. Homme, Mark R. Geier

**Affiliations:** 1 Institute of Chronic Illnesses, Inc., 14 Redgate Ct, Silver Spring, MD, USA; 2 Associate Professor of Biology, Simpson University, Redding, CA, USA; 3 CoMeD, Inc, Silver Spring, MD, USA; 4 International Academy of Oral Medicine and Toxicology, ChampionsGate, FL, USA

**Keywords:** ethylmercury, merthiolate, thiomersal, tic, tourette, vaccine

## Abstract

A hypothesis testing, case-control study evaluated automated medical records for exposure to organic-Hg from Thimerosal-containing hepatitis B vaccines (TM-HepB) administered at specific intervals in the first six-months-of-life among cases diagnosed with a tic disorder (TD) or cerebral degeneration (CD) (an outcome not biologically plausibly linked to TM exposure) in comparison to controls; both cases and controls were continuously enrolled from birth (born from 1991–2000) within the Vaccine Safety Datalink (VSD) database. TD cases were significantly more likely than controls to have received increased organic-Hg from TM-HepB administered within the first month-of-life (odds ratio (OR)=1.59, *p*<0.00001), first two-months-of-life (OR=1.59, *p*<0.00001), and first six-months-of-life (OR=2.97, *p*<0.00001). Male TD cases were significantly more likely than male controls to have received increased organic-Hg from TM-HepB administered within the first month-of-life (OR =1.65, *p*<0.0001), first two-months-of-life (OR=1.64, *p*<0.0001), and first six months-of-life (OR=2.47, *p*<0.05), where as female TD were significantly more likely than female controls to have received increased organic-Hg from TM-HepB administered within the first six-months-of-life (OR=4.97, *p*<0.05). By contrast, CD cases were no more likely than controls to have received increased organic-Hg exposure from TM-HepB administered at any period studied within the first six-months-of-life. Although routine childhood vaccination is considered an important public health tool to combat infectious diseases, the present study associates increasing organic-Hg exposure from TM-HepB and the subsequent risk of a TD diagnosis.

## Introduction

Tic disorder (TD) is a neurodevelopmental disorder characterized by repetitive, involuntary movements and vocalizations called tics (Diagnostic and Statistical Manual of Mental Disorders – Fifth Edition; DSM-5, 2013; Roessner *et al*., 2011). TD includes Tourette's syndrome, which is characterized by vocal as well as motor tics. Symptoms of TD typically begin in childhood, with the average onset between 3 and 9 years of age. Males are affected approximately three to four times more often than females. TD is considered a chronic condition that lasts a lifetime (National Institute of Neurological Disorders and Stroke, 2012). Psychopathology and co-morbidity occur in approximately 80–90% of clinical cohorts (Hariz *et al*., 2010). Two of the most common co-occurring psychiatric conditions are: (1) attention deficit/hyperactivity disorder (ADHD), occurring in about half the cases (Roessner *et al*., 2011; Freeman 2007) and (2) obsessive–compulsive disorder (OCD), also occurring in about half the cases (Roessner *et al*., 2011). Other common co-morbid conditions are depression, anxiety, and behavioral disorders (Hariz *et al*., 2010). Also reported are social difficulties and ritualistic behaviors such as counting, repeating, ordering, and arranging. Along with the dramatic rise in neurodevelopmental disorders in general in the last two decades, there has also been an increase in TD (Boyle *et al*., 2011; Cubo 2012). Although TD was once considered rare, today TD is considered the most common movement disorder, with 0.2–46.3% of schoolchildren experiencing tics during their lifetime (Cubo, 2012). To date, there is no consensus on the causes or contributing factors related to this increase. Many questions regarding the potential contribution of genetic inheritance and susceptibility, gene/environment interaction, and epigenetic or environmental factors to TD remain unanswered.

In 2000, investigators described the apparent first childhood case report of mercury (Hg) intoxication with tics as its only manifestation (Li *et al*., 2000). Subsequently, a series of epidemiological studies evaluated the potential relationship between exposure to organic-Hg from Thimerosal (TM) in childhood vaccines and the risk of a child being diagnosed with a TD (Thompson *et al*., 2007; Verstraeten *et al*., 2003; Andrews *et al*., 2004; Barile *et al*., 2012; Geier & Geier, 2005; Young *et al*., 2008). These previous studies employed various epidemiological methods such as case-control or cohort designs, and were conducted on children from several different countries. Each of these studies revealed a significant association between organic-Hg exposure from TM in childhood vaccines and the risk of a child being diagnosed with a TD, and several even observed a significant dose-dependent relationship between increasing organic-Hg exposure from TM-containing vaccines administered at specific intervals within the first year of life and the eventual risk of a child being diagnosed with a TD.

The purpose of the present study was to further evaluate the potential relationship between exposure to organic-Hg from TM-containing hepatitis B vaccines (TM-HepB) administered at specific intervals during the first six months of life, and the subsequent risk of a child being diagnosed with a TD, by conducting a case-control epidemiological study of automated medical records in the Vaccine Safety Datalink (VSD) database.

## Materials and methods

The study protocol employed was approved by the US Centers for Disease Control and Prevention (CDC), the Institutional Review Board (IRB) of Kaiser Permanente North-West (KPNW), and the IRB of Kaiser Permanente Northern California (KPNC). The data was analyzed at the secure Research Data Center of the National Center for Health Statistics in Hyattsville, MD. The views expressed in this study do not necessarily reflect those of the CDC or those of Kaiser Permanente.

The VSD project was created in 1991 by the National Immunization Program (NIP) of the CDC. The VSD's data collection and study methods have been previously described (Chen *et al*., 1997, 2000; Wassilak *et al*., 1995; Geier & Geier, 2004). The project links medical event information, specific vaccine history, and selected demographic information from the computerized databases of several health maintenance organizations (HMOs). The specific epidemiological methods of analysis employed in the present study were described in a recent study (Geier *et al*., 2013).

### Determining the population at risk

A cohort of over 1 million infants enrolled in the VSD project (updated through the end of 2000) from KPNW, Kaiser Permanente Colorado (KPC), and KPNC was examined using SAS^®^ software. The cohort examined was comprised of individuals who were HMO-enrolled from their date of birth and whose records specified their gender.

### Determining Cases

The outcome files (inpatient and outpatient diagnoses) from this population were then reviewed to find the first instance of TD for each child as defined by the International Classification of Disease, 9^th^ revision (ICD-9). The TD-associated ICD-9 diagnostic codes examined included: tic disorder, unspecified (307.20), transient tic disorder (307.21), chronic motor or vocal tic disorder (307.22), and Tourette's disorder (307.23). If there were multiple instances of the same diagnosis in a child, only the first instance was counted. In addition, to ensure that exposure preceded the diagnosis of a TD, for vaccinated individuals diagnosed with a TD, only those individuals diagnosed with a TD following administration of the vaccines under study were included as cases in the present analyses (less than 10% of individuals diagnosed with a TD were excluded because of this criterion).

A total of 344 cases diagnosed with TD (males = 253, females = 91, male/female ratio = 2.8), born from 1991 through 2000, were identified. These individuals diagnosed with TD were evaluated to determine their mean age of initial diagnosis of TD ± standard deviation of mean age of initial diagnosis of TD (5.10±2.01 years-old).

In addition, the putative generally accepted biologically non-plausible linkage between TM exposure and a subsequent diagnosis of cerebral degeneration was examined as a control outcome. The outcome files (inpatient and outpatient diagnoses) from this population were reviewed to find the first instance of cerebral degeneration for each child defined as 330.xx or 331.xx by ICD-9 coding. If there were multiple instances of the same diagnosis in a child, only the first instance was counted. In addition, to ensure the potential for an association between exposure and outcome, for vaccinated individuals diagnosed with cerebral degeneration, only those individuals diagnosed with cerebral degeneration following administration of the vaccines under study were included as cases in the present analyses.

A total of 647 cases diagnosed with cerebral degeneration (males = 359, females = 288, male/female ratio = 1.25), born from 1991 through 2000, were identified. These individuals diagnosed with cerebral degeneration were evaluated to determine their mean age of initial diagnosis of TD ± standard error of mean age of initial diagnosis of cerebral degeneration (0.63±0.04 years-old).

### Determining controls

In order to identify controls without a diagnosis of TD who would have only a minimal chance of subsequently receiving such a diagnosis, controls had to have been continuously enrolled from birth for at least 7.11 years (mean age of initial diagnosis of TD plus the standard deviation of mean age of initial diagnosis of TD). Applying this follow-up criterion yielded a total of 28,016 controls without a TD diagnoses (males = 14,327, females = 13,689, male/female ratio = 1.05) born from 1991 through 1993.

In order to identify controls without a diagnosis of cerebral degeneration who would have only a minimal chance of subsequently receiving such a diagnosis, controls had to have been continuously enrolled from birth for at least 2.6 years (mean age of initial diagnosis of cerebral degeneration plus two times the standard deviation of mean age of initial diagnosis of cerebral degeneration). Applying this follow-up criterion yielded a total of 135,888 controls without a cerebral degeneration diagnoses (males = 69,426, females = 66,462, male/female ratio = 1.05) born from 1991 through 1998.

### Hepatitis B vaccine exposure

The vaccine file for cases and controls was then reviewed to determine the exact dates of HepB administration. Those cases and controls receiving no doses of HepB were also included in the present study. Hg exposure was assigned as 12.5 microgram (μg) of organic-Hg per dose for those receiving a pediatric HepB or 0 μg of organic-Hg per dose for those receiving either combined haemophilus influenzae type b (Hib)-HepB or neither of the aforementioned vaccines. The Hg content of the vaccine doses was based upon the report of the Committee on Infectious Disease and Committee on Environmental Health of the American Academy of Pediatrics report of the TM content in US-licensed vaccines from 1999 (American Academy of Pediatrics 1999). Overall among the cases and controls, the maximum exposure to Hg from pediatric HepB was 37.5 μg of organic-Hg (from children receiving three doses of TM-HepB) administered within the first six months of life.

### Statistical analyses

The Fisher's exact test contained in the SAS^®^ software was utilized for statistical analyses, and a two-sided *p*-value <0.05 was considered statistically significant. Three different levels of Hg exposure from TM-HepB were examined. In the first case-control experimental group (Experiment I), the data were examined to determine the frequency of exposure to 12.5 μg of organic-Hg from TM-HepB in the first month of life, in comparison to the frequency of 0 μg of organic-Hg from TM-free hepatitis B vaccine (TM-free-HepB) or no HepB in the first month of life, among cases and controls. In the second case-control experimental group (Experiment II), the data were examined to determine the frequency of receiving two TM-HepB within the first two months of life or a total of 25 μg of organic-Hg, in comparison to the frequency of receiving 0 μg of organic-Hg from TM-free-HepB and/or no HepB in the first two months of life, among cases and controls. In the third case-control experimental group (Experiment III), the data were examined to determine the frequency of receiving three TM-HepB within the first six months of life, or a total of 37.5 μg of organic-Hg, in comparison to the frequency of receiving 0 μg of organic-Hg in the first sixth months of life, from TM-free-HepB and/or no HepB among cases and controls. In addition, because the ratio of males to females was 2.8, using the aforementioned exposures and exposure windows, additional separate analyses were completed where male cases were compared to male controls (Experiments IV–VI) and female cases were compared to female controls (Experiments VII–IX). Finally, using the aforementioned exposures and exposure windows, cases diagnosed with the putative non-biological plausibly linked TM-associated diagnosis of cerebral degeneration, a medical condition that is generally accepted as biologically not plausibly linked to TM exposure was compared to controls (Experiments X–XII). The overall null hypothesis for each of these case-control experimental groups was that there would be no difference in the frequency of exposure to organic-Hg doses from TM-HepB between the cases and the controls.

### Results

Table 1 displays the relationship between cases diagnosed with a TD and controls receiving increasing doses of organic-Hg from TM-HepB at several specific points within the first six months of life. Experiment I documented that cases diagnosed with TD were significantly more likely (odds ratio = 1.59, *p*<0.0001) than controls to have received 12.5 μg of organic-Hg from TM-HepB in comparison to 0 μg of organic-Hg from TM-free-HepB or no HepB within the first month of life. Experiment II documented that cases diagnosed with a TD were significantly more likely (odds ratio = 1.59, *p*<0.0001) than controls to have received 25 μg of organic-Hg from TM-HepB in comparison to 0 μg of organic-Hg from TM-free-HepB and/or no HepB within the first two months of life. Finally, in Experiment III, cases diagnosed with a TD were significantly more likely (odds ratio = 2.97, *p*<0.005) than controls to have received 37.5 μg of organic- Hg from TM-HepB in comparison to 0 μg of organic-Hg from TM-free-HepB and/or no HepB within the first six months of life.

**Table 1:** A summary of exposure to organic-Hg from Thimerosal-containing hepatitis B vaccine administration among cases diagnosed with TD in comparison to controls.

Group Examined		Number of Cases Diagnosed with a TD (%)	Number of Controls without a TD Diagnosis (%)	Odds Ratio (95% CI)	*p*-value
Experiment I	12.5 μg organic-Hg within 1st month	151 (43.90)	9,222 (32.92)	1.59 (1.29–1.98)	<0.00001
	0 μg organic-Hg within 1st month	193 (56.10)	18,794 (67.08)		
Experiment II	25 μg organic-Hg within first 2 months	151 (44.54)	9,236 (33.62)	1.59 (1.28–1.97)	<0.00001
	0 μg organic-Hg within first 2 months	188 (55.46)	18,233 (66.38)		
Experiment III	37.5 μg organic-Hg within first 6 months	33 (76.74)	1,292 (52.63)	2.97 (1.46–6.05)	0.005
	0 μg organic-Hg within first 6 months	10 (23.26)	1,163 (47.37)		

Tables 2 and 3 present the relationship between males cases diagnosed with a TD in comparison to male controls and female cases diagnosed with a TD in comparison to female controls receiving increasing doses of organic-Hg from TM-HepB at several specific points within the first six months of life. Experiment IV documented that male cases diagnosed with a TD were significantly more likely (odds ratio = 1.65, *p*<0.001) than male controls to have received 12.5 μg of organic-Hg from TM-HepB in comparison to 0 μg of organic-Hg from TM-free-HepB or no HepB within the first month of life. Experiment V documented that male cases diagnosed with a TD were significantly more likely (odds ratio = 1.64, *p*<0.001) than male controls to have received 25 μg of organic-Hg from TM-HepB in comparison to 0 μg of organic-Hg from TM-free-HepB and/or no HepB within the first two months of life. Finally, in Experiment VI, male cases diagnosed with a TD were significantly more likely (odds ratio = 2.47, *p*<0.05) than male controls to have received 37.5 μg of organic-Hg from TM-HepB in comparison to 0 μg of organic-Hg from TM-free-HepB and/or no HepB within the first six months of life. By contrast, only females diagnosed with a TD in comparison to female controls were significantly more likely (odds ratio = 4.97, *p*<0.05) to have received 37.5 μg of organic-Hg from TM-HepB in comparison to 0 μg of organic-Hg from TM-free-HepB and/or no HepB within the first six months of life (Experiment IX).

**Table 2:** A summary of exposure to organic-Hg from Thimerosal-containing hepatitis B vaccine administration among male cases diagnosed with TD in comparison to male controls.

Group Examined		Number of Male Cases Diagnosed with TD (%)	Number of Male Controls without a TD Diagnosis (%)	Odds Ratio (95% CI)	*p*-value
Experiment IV	12.5 μg organic mercury within 1st month	113 (44.66)	4,697 (32.78)	1.65 (1.29–2.12)	<0.0001
	0 μg organic mercury within 1st month	140 (55.34)	9,630 (67.22)		
Experiment V	25 μg organic mercury within first 2 months	113 (45.20)	4,704 (33.51)	1.64 (1.27–2.10)	<0.0001
	0 μg organic mercury within first 2 months	137 (54.80)	9,333 (66.49)		
Experiment VI	37.5 μg organic mercury within first 6 months	22 (73.33)	640 (52.72)	2.47 (1.09–5.58)	<0.05
	0 μg organic mercury within first 6 months	8 (26.67)	574 (47.28)		

**Table 3:** A summary of exposure to organic-Hg from Thimerosal-containing hepatitis B vaccine administration among female cases diagnosed with TD in comparison to female controls.

Group Examined		Number of Female Cases Diagnosed with TD (%)	Number of Female Controls without a TD Diagnosis (%)	Odds Ratio (95% CI)	*p*-value
Experiment VII	12.5 μg organic mercury within 1st month	38 (41.76)	4,525 (33.06)	1.45 (0.95–2.21)	0.09
	0 μg organic mercury within 1st month	53 (58.24)	9,164 (66.94)		
Experiment VIII	25 μg organic mercury within first 2 months	38 (42.70)	4,532 (33.74)	1.46 (0.96–2.23)	0.09
	0 μg organic mercury within first 2 months	51 (57.30)	8,900 (66.26)		
Experiment IX	37.5 μg organic mercury within first 6 months	11 (84.61)	652 (52.54)	4.97 (1.1–22.5)	< 0.05
	0 μg organic mercury within first 6 months	2 (15.39)	589 (47.46)		

Table 4 summarizes the relationship between cases diagnosed with the cerebral degeneration, a medical condition that is not felt to be biologically linked to TM exposure, in comparison to controls receiving increasing doses of organic-Hg from TM-HepB at several specific points within the first six months of life. The results revealed that cases diagnosed with cerebral degeneration in comparison to controls were actually significantly less likely to receive increasing doses of organic-Hg from TM-HepB in comparison to 0 μg of organic-Hg from TM-free-HepB and/or no HepB within the first, second, and sixth months of life (Experiments X–XII).

**Table 4:** A summary of exposure to organic-Hg from Thimerosal-containing hepatitis B vaccine administration among cases diagnosed with cerebral degeneration in comparison to controls.

Group Examined		Number of Cases Diagnosed with Cerebral Degeneration (%)	Number of Controls without a Cerebral Degeneration Diagnosis (%)	Odds Ratio (95% CI)	p-value
Experiment X	12.5 μg organic-Hg within 1st month	175 (27.05)	62,637 (46.09)	0.43 (0.36–0.52)	<0.00001
	0 μg organic-Hg within 1st month	472 (72.95)	73,252 (53.91)		
Experiment XI	25 μg organic-Hg within first 2 months	172 (27.13)	62,637 (46.63)	0.42 (0.36–0.51)	<0.00001
	0 μg organic-Hg within first 2 months	462 (72.87)	71,694 (53.37)		
Experiment XII	37.5 μg organic-Hg within first 6 months	59 (39.07)	13,145 (71.86)	0.25 (0.18–0.35)	<0.005
	0 μg organic-Hg within first 6 months	92 (60.93)	5,148 (28.14)		

### Discussion

Consistent with the aforementioned six epidemiological studies that found a significant relationship between organic-Hg exposure from TM in childhood vaccines and the risk of a child being diagnosed with a TD (Thompson *et al*., 2007; Verstraeten *et al*., 2003; Andrews *et al*., 2004; Barile *et al*., 2012; Geier & Geier 2005; Young *et al*., 2008), the results from the present study show a significant association between increased organic-Hg exposure from TM-HepB administered at specific interval within the first six months of life and the eventual risk of child receiving a TD diagnosis. Also, consistent with the aforementioned epidemiological studies that showed a dose-dependent relationship between increasing organic-Hg exposure from TM in childhood vaccines and increasing risk of a child being diagnosed with a TD, the results of the present study showed that increased organic-Hg exposure from TM-HepB was associated with an increased eventual risk of a child receiving a TD diagnosis.

The biological plausibility of a significant association between organic-Hg exposure from TM in childhood vaccines and the risk of a TD diagnosis is supported by the fact that TM is known to rapidly dissociate into ethyl-Hg chloride, ethyl-Hg hydroxide, and sodium thiosalicylate in saline solutions (Tan & Parkin, 2000). In addition, it was observed in human infants that administering TM-containing vaccines significantly increased an infant's blood- and hair-Hg levels, with some infants having total blood- and hair-Hg levels in excess of the safety limit adopted by the US Environmental Protection Agency (EPA) (Stajich *et al*., 2000; Pichichero *et al*., 2008, 2009; Redwood *et al*., 2001; Marques *et al*., 2007). It was even observed by investigators that TM administration to infant monkeys mimicking the US early childhood vaccination schedule of the 1990s resulted in significant levels of Hg being present in the brain (Burbacher *et al*., 2005), and that ethyl-Hg species are actively transported across neuronal cellular membranes (Zimmermann *et al*., 2013; Wehe *et al*., 2014).

Once any form of Hg enters the brain, it immediately has a plethora of negative effects, one of which is axonal degeneration, particularly of large caliber axons that tend to connect distant parts of the brain (Kern *et al*., 2012, 2013). Unfortunately, once these long-range axons are destroyed, they are unlikely to be regenerated. In other words, the shorter the distance between the regeneration site and its distal target, the more successful the regeneration of a nerve is likely to be (Fawcett, 1992). Overall, postnatally damaged mature neuronal axons only regenerate for very short distances in the central nervous system (CNS). Because long-range axons cannot be regenerated, short-range axons result when long-range axons are destroyed. It has been shown that following damage to connected brain regions, the brain undergoes an adaptive response which includes reactive axonal sprouting and an overproduction of dendrites (Jones, 1999; Jones *et al*., 1992).

To this point, researchers have shown long-range axon loss (with subsequent long-range underconnectivity) along with short-range overconnectivity in subjects diagnosed with a TD (Church *et al*., 2009; Liu *et al*., 2013; Neuner *et al*., 2010). Church *et al*. (2009), for example, examined functional connectivity in subjects diagnosed with a TD and theorized that the decreased long-range functional connectivity between control regions in the brain *(e.g.,* between the dorsolateral prefrontal cortex and the posterior parietal cortex), along with the short-range overconnectivity *(e.g.,* between the anterior and the dorsolateral prefrontal cortices) may explain the inability to control unwanted behaviors in subjects diagnosed with a TD.

This evidence of long-range underconnectivity and short-range overconnectivity found in subjects diagnosed with a TD is also characteristic of ADHD and autism spectrum disorder (ASD) (Fair *et al*., 2007; Wang *et al*., 2009; Castellanos *et al*., 2008; Wass, 2011). In addition, TD shares additional symptomatology with ADHD and ASD, including issues with attention, impulsivity, repetitive behaviors, social impairment, anxiety, obsessive compulsive behaviors, and depression (Grzadzinski *et al*., 2011; Kern *et al*., 2015; National Institute of Neurological Disorders and Stroke, 2013; Hariz *et al*., 2010). Finally, consistent with the significant association observed between organic-Hg exposure from TM-containing vaccine administration and the risk of a child being diagnosed with a TD (Thompson *et al*., 2007; Verstraeten *et al*., 2003; Andrews *et al*., 2004; Barile *et al*., 2012; Geier & Geier, 2005; Young *et al*., 2008), it was observed in a series of previous epidemiological studies that a significant association was observed between organic-Hg exposure from TM-containing vaccine administration at specific intervals within the first year of life and the eventual risk of child being diagnosed with ADHD or ASD (Young *et al*., 2008; Geier & Geier, 2005, 2006; Geier *et al*., 2013).

### Strengths/limitations

As described by investigators from the CDC and US Food and Drug Administration (FDA), products, such as vaccines, which are intended as disease-preventive interventions given to healthy people, must be held to a high standard of safety assurance (Ellenberg & Braun, 2002; DeStefano *et al*., 2001). However, those investigators reported that the study of vaccine risks is more complex than for therapeutic products because exposure is virtually universal for many vaccines, ensuring the chance occurrence of many adverse outcomes in temporal association with vaccines. As a result, those investigators identified the utility of using the VSD, a consortium of HMOs to rigorously evaluate vaccine-associated risks in hypothesis testing studies.

The strength of the VSD is revealed in the present study because the observations made were based on a retrospective assessment of prospectively collected medical records. Any potential independent variables that might have been associated with either enrollment or healthcare-seeking behaviors were moot because all cases were required to be enrolled from birth until a TD was diagnosed, and all controls had to be enrolled from birth for a time period sufficient to minimize the chance that a TD diagnosis would emerge during follow-up. In addition, vaccinated cases diagnosed with a TD were specifically evaluated to ensure that only those vaccinated cases diagnosed with a TD following their HepB vaccine administration were considered in the present analyses.

That the VSD data were collected independently of the present study is another strength of the study. The VSD data records were collected as part of the routine healthcare that patients received through participation with their respective HMOs, and as such, the healthcare providers were not contemplating any possible associations between vaccine exposures and potential health outcomes.

An additional strength of the present study was that the specific methods employed to evaluate the hypothesis were also able to exploit recommendations for the timing of vaccine administration that varied widely. Specifically, differences in cumulative doses of organic-Hg received at specific intervals during the infant period were evaluated based upon the wide-ranging recommendations for routine HepB administration. In 1991, the Advisory Committee on Immunization Practices (ACIP) recommended that infants should receive their HepB doses as follows: first dose between birth and 2 months of age, second dose between 1 and 4 months of age, and third dose between 6 and 18 months of age (CDC, 1991). Importantly, the differences in organic-Hg exposures observed in all experiments of the present study were not the result of a small group of children receiving anomalous exposures to vaccines. Instead, the experiments assessed varying levels of organic-Hg exposure which resulted from the varying windows recommended for administration of HepB during the first 18 months of life.

A further strength of the present study was that adequate follow-up was employed to ensure that most subjects in the control group were unlikely to be subsequently diagnosed with a TD. This was achieved by setting an *a priori* requirement that, to be a valid control-cohort member, a child had to be continuously enrolled in the VSD from birth until the child attained the mean age of initial TD diagnosis plus the standard deviation for that mean age, which was 7.11 years of age based on the distribution statistics for the cases. Statistically setting the follow-up age for the control group in this manner assured a less than a 16% chance that some of the controls might subsequently be diagnosed with a TD. Ideally, a longer follow-up period would have further reduced this misclassification risk. However, the limitations on the VSD patient data records that were available for review precluded a longer follow up period because the VSD data available for examination ended in 2000. It is important to ensure that most controls will have little risk of subsequently being diagnosed, because for outcomes that have a wide onset window, like TD, the statistical “noise” in the exposure signal will be minimized when a sufficient follow-up is ensured.

By reducing the length of follow-up, and hence introducing greater uncertainty as to the correct diagnostic status of the controls, the magnitude of the observed adverse effects associated with exposure would be reduced, which in turn would bias the results toward the null hypothesis. For example, by requiring that those in the control group only having to be continuously enrolled in the VSD from birth until they were at least 5.10 years-old (the mean age of initial TD diagnosis), those in the TD case group were no longer significantly more likely (odds ratio = 1.42, *p*<0.40) than controls to have received 37.5 μg of organic-Hg from TM-HepB within the first six months of life than 0 μg of organic-Hg from TM-free-HepB in the first six months of life.

The results of the present study may have a number of potential limitations. It is theoretically possible that the observed results may have occurred from unknown biases or confounders present in the datasets examined. However, this seems unlikely because the putative biologically non-plausible linkage between TM exposure and a subsequent diagnosis of cerebral degeneration was examined as a control outcome, using the same VSD database and methodology employed for TD. No similar patterns of significant associations were observed for these outcomes. Moreover, the diagnosed cases having TM exposure that is generally accepted as being not biologically linked to a diagnosis cerebral degeneration were significantly less likely than controls to receive increasing organic-Hg exposure in comparison to TM-free-HepB or no HepB administered at specific intervals within the first six months of life. The observed apparent protective effect of TM on the risk of an individual being diagnosed with cerebral degeneration suggests that a “healthy vaccine effect” was present in our data. As described previously by investigators from the CDC (Fine & Chen, 1992), confounding of this sort is a general problem for studies of adverse reactions to prophylactic interventions, as they may be withheld from some individuals precisely because they are already at high risk of the adverse event, and as consequence studies that fail to control adequately for such confounding factors are likely to underestimate the risk of adverse events attributable to vaccination. Since this phenomenon is apparent in the data examined, the significantly increased odds ratios observed for increasing organic-Hg exposure among cases diagnosed with TD are probably underestimates of the true extent of the relationship between organic-Hg exposure from TM and the TD outcome studied.

Another theoretical limitation of the present study is that the results observed for TD may be due to statistical chance. However, such a possibility would be unlikely given the limited number of statistical tests performed, the highly significant results observed, and the consistency in the direction and magnitude of the results observed.

Still other theoretical limitations of the present study include the possibilities that some of the children in the cohorts may have had subtle neurological dysfunction that was not recorded in the medical record; healthcare providers may have misdiagnosed some individuals; or some vaccine exposures may not have been appropriately classified. These possibilities should not have affected the results significantly because both cases and controls should have been affected similarly. Moreover, misclassification would tend to bias the results toward the null hypothesis, since such effects would result in individuals being placed in the wrong exposure and/or outcome categories, and this would result in decreased statistical power to determine true potential exposure-outcome relationships.

Another potential limitation of the present study is that exposures to other sources of Hg were not evaluated. The children examined in the present study very likely incurred other exposures to Hg from other TM-containing vaccines, breastfeeding, formula feeding, and, possibly, dental amalgams, fish, or other environmental sources. The limitation of not being able to associate other vaccine-related Hg exposures was one of the limitations specifically imposed by the CDC staff who assembled our VSD dataset. While other sources of Hg may play a significant role in the pathogenesis of TD, such exposures, which were not accounted for in this study, would actually tend to bias the results towards the null hypothesis by confounding the Hg exposure classifications examined. For example, individuals classified as having lower organic-Hg exposure from TM-containing vaccines may have actually received high doses of Hg from other sources, and individuals having higher organic-Hg exposure from TM-containing vaccines may have actually received low doses of Hg from other sources, with the net result tending to minimize the magnitude of the associations observed.

An additional potential limitation of the present study is that the individuals examined participated with different HMOs, and, as such, HMO differences may account for some of the phenomena observed. This phenomena would seem to be unlikely because each HMO studied followed similar national ACIP guidelines for HepB vaccine administration and used similar ICD-9 coding to medically document the outcomes of individuals. In addition, because of the manner in which the CDC staff provided us our VSD datasets, it was not possible to identify the specific HMOs that each individual was enrolled. Despite this limitation in our VSD datasets, we believe that it would not necessarily be prudent to stratify the data by HMO, because to do so would assume that the differences in population sizes for each HMO should be given equal weight with respect to their potential impact on the phenomena observed, despite each HMO having varying degrees of statistical power to detect the phenomena observed based upon the size of the HMO population.

Finally, the current study suffers from the potential limitation that the analyses were not conducted to explore the precise timing and cumulative doses of organic-Hg from all TM-containing childhood vaccines associated with maximum potential adverse consequences. In future studies, it would be worthwhile to explore these precise timing and cumulative-dose phenomena. In addition, evaluating other neurodevelopmental outcomes, as well as other covariates such as gender, race, birth weight, *etc.*, that may affect the magnitude of the adverse effects found, would be valuable.

## Conclusion

In summary, using a hypothesis-testing, epidemiological methodology applied to the VSD database, organic-Hg exposure from TM-containing childhood vaccines was determined to be a significant risk factor for the subsequent diagnosis of TD. Six previous epidemiological studies support this finding (Thompson *et al*., 2007; Verstraeten *et al*., 2003; Andrews *et al*., 2004; Barile *et al*., 2012; Geier & Geier, 2005; Young *et al*., 2008). In addition, this study, consistent with previous epidemiological studies, shows a dose dependent effect *(i.e.,* the greater the exposure to Hg, the greater the chances of having a TD diagnosis).

As mentioned previously, since Hg may accumulate in the brain and other tissues, the combined exposure from different sources is a serious concern, especially in the face of an underlying chronic environmental exposure that appears to be increasing (Kern *et al*., 2011; Laks, 2009). Additive effects from different sources could potentially increase a child’s Hg exposure from a subclinical to a clinical level, with neurodevelopmental consequences. Thus, every preventable source of Hg exposure should be evaluated and avoided. Routine childhood vaccination is considered an important public health tool to reduce the morbidity and mortality associated with infectious diseases (Geier & Geier, 2002). However, based on data, including the results of the present study that show an association between administration of a TM-containing vaccine and adverse neurodevelopmental outcomes, it is also a public health imperative to end the addition of organic-Hg to vaccines in the form of TM used as a preservative.
